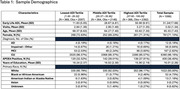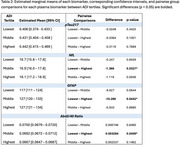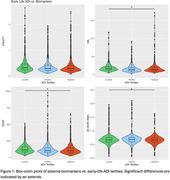# Associations between Early‐Life Area‐Level Disadvantage and Plasma Markers of Alzheimer’s Disease Pathology and Neurodegeneration – A Mixed‐Longitudinal Study

**DOI:** 10.1002/alz70861_107967

**Published:** 2025-12-23

**Authors:** Jason F Moody, Samriddhi Dube, William R. Buckingham, W. Ryan Powell, Sarah A. Keller, Amanda DeWitt, Rachael E. Wilson, Kaj Blennow, Henrik Zetterberg, Sterling C Johnson, Amy J.H. Kind, Barbara B. Bendlin

**Affiliations:** ^1^ Wisconsin Alzheimer's Disease Research Center, School of Medicine and Public Health, University of Wisconsin‐Madison, Madison, WI USA; ^2^ Center for Health Disparities Research, University of Wisconsin School of Medicine and Public Health, Madison, WI USA; ^3^ Health Services and Care Research Program, University of Wisconsin‐Madison, School of Medicine and Public Health, Madison, WI USA; ^4^ Wisconsin Alzheimer's Disease Research Center, University of Wisconsin‐Madison, School of Medicine and Public Health, Madison, WI USA; ^5^ Wisconsin Alzheimer's Institute, School of Medicine and Public Health, University of Wisconsin‐Madison, Madison, WI USA; ^6^ Department of Psychiatry and Neurochemistry, Institute of Neuroscience and Physiology, The Sahlgrenska Academy, University of Gothenburg, Mölndal Sweden; ^7^ Clinical Neurochemistry Laboratory, Sahlgrenska University Hospital, Mölndal, Västra Götalands län Sweden; ^8^ Hong Kong Center for Neurodegenerative Diseases, Hong Kong, Science Park China; ^9^ Department of Neurodegenerative Disease, National Hospital for Neurology and Neurosurgery, UCL Institute of Neurology, London UK; ^10^ UK Dementia Research Institute at UCL, London UK; ^11^ Wisconsin Alzheimer’s Disease Research Center, School of Medicine and Public Health, University of Wisconsin‐Madison, Madison, WI USA; ^12^ Wisconsin Alzheimer’s Institute, School of Medicine and Public Health, University of Wisconsin‐Madison, Madison, WI USA

## Abstract

**Background:**

Recent literature suggests that the cumulative social determinants of health (SDOH) of a specific geographic region may have a greater impact on health outcomes compared to individual‐level socioeconomic factors, including on the incidence of age‐related diseases. Research is needed into how area‐level disadvantage from across the life course, particularly early in life, may disproportionately affect Alzheimer’s disease (AD) pathology observed later in life. Plasma biomarkers have shown promise for providing a cost‐effective, non‐invasive means for detecting AD pathogenesis and tracking AD progression. In this study, we examine the associations between early‐life area‐level disadvantage and AD plasma biomarkers in aging adults.

**Methods:**

We recruited 1,099 participants across the AD continuum (Table 1) from one of two community‐based, longitudinal cohorts enriched for AD risk who had at least 1 plasma sample assayed for markers of amyloid plaques [plasma phosphorylated tau (pTau)217, amyloid beta (Aβ)_42/40_ ratio], neuroinflammation [glial fibrillary acidic protein (GFAP)], and axonal degeneration [neurofilament light (NfL)]. Early life area‐level disadvantage (ages 0‐18) was quantified via the county‐level Area Deprivation Index (ADI), a composite marker derived from 17 census indicators of SDOH, and categorized into sample‐ranked tertiles (lowest, middle, and highest). We used linear mixed‐effects (LME) models with early‐life ADI as the explanatory variable, adjusting for age, sex, impairment status, batch, and years of education, and included a random intercept to account for repeated, within‐subject measures. Group comparisons were performed for each biomarker between pairs of ADI tertiles, controlling for error rates across multiple comparisons.

**Results:**

Participants living in the highest ADI areas exhibited higher NfL [difference (p value): 1.37 (0.033)] and GFAP [difference (p value): 10.26 (0.044)] and lower Aβ_42/40_ [difference (p value): 0.00328 (0.041)], compared to those in the lowest ADI tertile. No significant relationships were observed between ADI tertiles and pTau217. (Table 2, Figure 1).

**Conclusion:**

Our findings suggest that addressing disparities in SDOH within the critical epoch of brain development encompassing infancy, childhood, and adolescence may hold the potential to reduce the likelihood of pathological brain changes associated with AD occurring later in life, including the accumulation of amyloid plaques, neuroinflammation, and axonal injury.